# A candidate gene association study on muscat flavor in grapevine (*Vitis vinifera *L.)

**DOI:** 10.1186/1471-2229-10-241

**Published:** 2010-11-09

**Authors:** Francesco Emanuelli, Juri Battilana, Laura Costantini, Loïc Le Cunff, Jean-Michel Boursiquot, Patrice This, Maria S Grando

**Affiliations:** 1 Research and Innovation Center, Fondazione Edmund Mach-Istituto Agrario di San Michele all'Adige, Via E. Mach 1, 38010 San Michele all'Adige (TN), Italy; 2 INRA UMR 1097 Diversité et Adaptation des Plantes Cultivées, 2 place Viala, 34060, Montpellier, France; 3 UMT GenoVigne®, IFV-INRA-Montpellier Supagro, 2 place Viala, 34060, Montpellier, France

## Abstract

**Background:**

The sweet, floral flavor typical of Muscat varieties (Muscats), due to high levels of monoterpenoids (geraniol, linalool and nerol), is highly distinct and has been greatly appreciated both in table grapes and in wine since ancient times. Muscat flavor determination in grape (*Vitis vinifera *L.) has up to now been studied by evaluating monoterpenoid levels through QTL analysis. These studies have revealed co-localization of 1-deoxy-D-xylulose 5-phosphate synthase (*VvDXS*) with the major QTL positioned on chromosome 5.

**Results:**

We resequenced *VvDXS *in an *ad hoc *association population of 148 grape varieties, which included muscat-flavored, aromatic and neutral accessions as well as muscat-like aromatic mutants and non-aromatic offsprings of Muscats. Gene nucleotide diversity and intragenic linkage disequilibrium (LD) were evaluated. Structured association analysis revealed three SNPs in moderate LD to be significantly associated with muscat-flavored varieties. We identified a putative causal SNP responsible for a predicted non-neutral substitution and we discuss its possible implications for flavor metabolism. Network analysis revealed a major star-shaped cluster of reconstructed haplotypes unique to muscat-flavored varieties. Moreover, muscat-like aromatic mutants displayed unique non-synonymous mutations near the mutated site of Muscat genotypes.

**Conclusions:**

This study is a crucial step forward in understanding the genetic regulation of muscat flavor in grapevine and it also sheds light on the domestication history of Muscats. *VvDXS *appears to be a possible human-selected locus in grapevine domestication and post-domestication. The putative causal SNP identified in Muscat varieties as well as the unique mutations identifying the muscat-like aromatic mutants under study may be immediately applied in marker-assisted breeding programs aimed at enhancing fragrance and aroma complexity respectively in table grape and wine cultivars.

## Background

Fragrance in table grapes and a persistent and complex aroma in wine are both sought after by the modern consumer. In particular, the floral flavor typical of Muscat varieties (also known as Muscats) is highly distinct and has been greatly appreciated since ancient times. Muscat vines are thought to be one of the oldest domesticated grapevines (*Vitis vinifera *L.) and they are now widely distributed all over the world [1]. It has been assumed that Muscats originated in Greece, the putative main progenitors of this large grape family being Moscato Bianco and Muscat of Alexandria [2]. Several studies have shown that the unique scent of muscat-flavored grape varieties is linked to the presence of monoterpenoids with a low olfactory perception threshold in the grape berry. In particular, linalool, geraniol, nerol, citronellol and α-terpineol have been described as the major aromatic determinants because of their high concentrations in Muscat cultivars [3,4]. Mateo and Jiménez [5] proposed a general classification of grape varieties based on monoterpene concentrations: a first group of intensely muscat-flavored varieties with a free monoterpene concentration as high as 6 mg/l (i.e. Muscat of Alexandria, Moscato Bianco, Gewürztraminer etc.); a second group of non-muscat but aromatic varieties with a total monoterpene concentration of 1-4 mg/l (i.e. Morio Muskat, Rhine Riesling, Sylvaner etc.) and a third group of more neutral varieties which do not depend upon monoterpenes for their flavor (i.e. Chardonnay, Chasselas, Cabernet-Sauvignon etc.). Monoterpenoids belong to the family of terpenoids, one of the most abundant and structurally diverse groups of natural metabolites essential for several biological functions of both primary and secondary metabolism [6]. Two distinct and partially independent routes, the cytoplasmatic mevalonic acid (MVA) pathway and the plastidial 2-methyl-D-erythritol-4-phosphate (MEP) pathway, have been identified in plants as producing isopentenyl diphosphate (IPP) and its isomer dimethylallyl diphosphate (DMAPP), the precursors of all terpenoids [7]. However, it is assumed that the MEP pathway is the dominant route for the biosynthesis of substrates of monoterpenes in the grape berry [8]. IPP and DMAPP are then condensed by the action of the prenyltransferase geranyldiphosphate synthase to yield geranyl diphosphate (GPP). Different monoterpene synthases subsequently catalyze the conversion of GPP to different cyclic and acyclic monoterpenoids. The primary monoterpene skeleton can be further modified by the action of various enzymes (i.e. cytochrome P450 hydroxylases, dehydrogenases and glycosyl and methyltransferases) [9,10].

The genetic bases of muscat flavor in grapevine have up to now been evaluated through QTL studies in distinct F1 biparental mapping populations [11,12] and in selfing populations [13]. Two major QTLs were confirmed in all the experiments, thus strengthening the hypothesis that muscat flavor determination is controlled by a reduced number of loci having a strong effect [14]. Doligez et al. [11] described the co-localization on linkage group (LG) 5 of the QTL for muscat flavor based on tasting data with a major QTL for monoterpenic odorant content. Battilana et al. [12] subsequently reported a positional candidate gene (CG), 1-deoxy-D-xylulose-5-phosphate synthase class 1 *(DXS)*, within the major QTL for the content of volatile and non-volatile forms of geraniol, nerol and linalool on LG 5.

DXS catalyzes the first reaction of the MEP pathway, the production of 1-deoxy-D-xylulose-5-phosphate (DXP) from the central metabolic intermediates glyceraldehyde 3-phosphate and pyruvate. Many investigations support a regulatory role for DXS in terpene biosynthesis in bacteria and in several plant species [15]. DXS regulation has been observed in plants both at the transcriptional level [16-18] and at the post-transcriptional level [19-21]. Accordingly, DXS was described as also being one of the main regulators of monoterpenoid biosynthesis in grapevine by Luan and Wüst [8]. The crucial role of DXS in regulating the MEP pathway is confirmed by altered phenotypes in *Arabidopsis *mutants *cla1-1, chs5, and lvr111 *due to a drastic decrease in chlorophyll content [22-24]. A small *DXS *gene family has been suggested for several plant species, i.e. *Arabidopsis thaliana *[15,25], *Ginko biloba *[26], great morinda [27], *Medicago truncatula *[28], oil palm [29], *Picea abies *[17], and *Pinus densiflora *[30]. Two or three potential *DXS*-like genes (*DXL*) have been reported in all these plants and phylogenetic analysis shows that these genes cluster into independent clades [30,31]. *DXL *genes display particular expression patterns suggesting a housekeeping function for *DXS *and tissue-specific roles in secondary isoprenoid biosynthesis for *DXL1 *and *DXL2 *[26,28,29,31,32]. One *DXS *(*DXS1*) located on chromosome 5, three *DXL1 *(*DXS2A, DXS2B *and *DXS2C*) located respectively on chromosomes 15, 11 and 7, and one *DXL2 *(*DXS3*) on chromosome 4 have been predicted in the grape genome [12].

In recent years, structured association (SA) mapping has emerged as a major tool in the search for the genes underlying quantitative trait variation in model plants [33,34] and other perennial plants [35]. Although genome-wide association (GWA) studies have recently gained preeminence [36,37], candidate gene association (CG) studies remain the key approach to gene mapping in less complex traits [38,39]. The extensive information obtained with the sequencing of the grape genome [40,41], and the definition of core collections retaining a high percentage of the genetic variability of natural collections [42] make GWA and CG association studies feasible in grapevine as well. The degree of LD, which is highly population-specific [43,44] and locus-specific [45], will determine the resolution of an association study, thus influencing the choice between CG or GWA strategies. Cultivated grapevine (*V. vinifera *subsp. sativa) has extensive genetic variation with a high level of long-range LD [46] making a GWA strategy feasible. On the other hand, intragenic LD decays rapidly in grapevine [47,48], favoring CG association approaches, as in the case of *Myb*-like genes tested for association with anthocyanin variation and berry color [49,50].

In the present study, we assessed the association of nucleotide variation in the candidate gene *VvDXS *with muscat flavor in grapevines with different genetic backgrounds. In order to avoid spurious associations, an SA analysis was carried out by testing individual polymorphic sites in one *ad hoc *association population incorporating the genetic structure of the sample as a covariate.

The objectives of the present study were to: (1) examine nucleotide diversity and LD within the *VvDXS *gene, (2) test for associations between individual polymorphisms and muscat flavor in order to identify putative causal SNPs, and (3) estimate the putative selection on this gene by calculating diversity index as Tajima's D and Fu and Li's D* and by performing a network analysis of reconstructed haplotypes. Possible implications for metabolic functions of the putative causal SNPs detected in muscat-flavored varieties and in muscat-like aromatic mutants are put forward and discussed. Moreover, the presence of a population structure in the dataset under study and the results of the network analysis are discussed with regard to the history of the domestication of cultivated grapevine and the transmission of muscat flavor.

## Results

### Validation of the candidate gene *VvDXS *expression into Muscat genetic background

**Figure 1:**
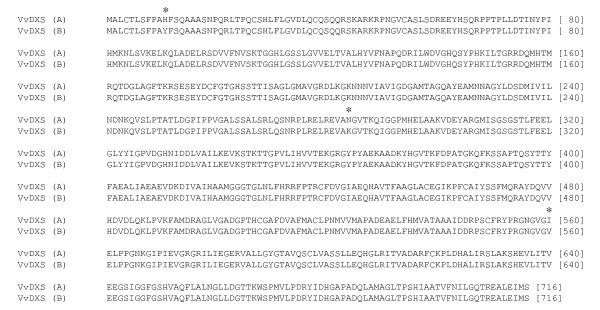
**VvDXS protein sequences predicted from full-ORF cDNA alleles of Moscato Bianco**. (A = allele A; B = allele B). * = non-synonymous changes; H11Y was not considered for the statistical analysis. Nexus (PAUP 3.0/MacClade) format was exported by MEGA 4 software, and amino acid positions are shown as numbers between brackets [ ].

In order to determine if the candidate gene *VvDXS *was expressed in the grape berry of Moscato Bianco, we amplified the full-ORF *VvDXS *cDNA from the cDNA retrotranscribed from total RNA of berry skin. The full-ORF *VvDXS *cDNA was then cloned and sequenced for an overall length of 2151 bp. Two *VvDXS *alleles could be distinguished and were defined as A and B based on a point mutation G/T (SNP 1822). VvDXS protein sequences of 716 amino acids for both Moscato Bianco alleles were predicted from the sequenced cDNA and were aligned (Figure 1).

### Description and nucleotide diversity of the candidate gene *VvDXS*

Candidate gene structure and nucleotide variation observed through analysis of 4790 bp of the *VvDXS *genomic sequence among the 148 grapevine genotypes is summarized in Table 1. *VvDXS *gene was split into 10 exons and 9 introns, and this structure corresponded to the gene prediction LOC 100249323 of *V. vinifera *PN40024. A total of 94 SNPs and 7 INDELs were identified and then named and scored according to their position on *VvDXS *ORF of *V. vinifera *PN40024. As *VvDXS *is predicted on the minus strand of locus NC_012011, nucleotide positions relative to NC_012011 are also reported (Additional file 1). SNP variation among the 148 grapevine accessions corresponded to an average of one SNP every 51 bp. As would be expected, the frequency of sequence variants was higher in non-coding regions (one every 38 bp) than in coding regions (one every 86 bp). A 1.5:1 ratio of synonymous to non-synonymous changes was observed in coding regions. INDEL frequency corresponded to one every 670 bp and INDELs were found only in introns as mononucleotide (5), dinucleotide (1) and 36 bp (1) variants.

**Table 1:** Nucleotide diversity of *VvDXS *in grapevine

Parameters	Overall	Coding/non coding
Varieties	148	
Genomic ATG-TAG (protein coding region)	4790 bp	
Full-ORF cDNA	2151 bp	
Predicted protein	716 aa	
Exons	10	
Introns	9	
Number of polymorphic sites	101	
SNPs	95	25/70
INDELS	7	0/7
Frequency of SNPs	1 per 51 bp	1 per 86/1 per 38
Frequency of INDELS	1 per 670 bp	-/1 per 372 bp
Synonymous changes	15	
Non-synonymous changes	10	
Synonymous vs non-synonymous mutations	1.5:1	

#### Nucleotide diversity

Nucleotide diversity (π = 0.0032, θ = 0.0034) was not equally distributed among site categories. The estimated π value was on average four times higher for synonymous sites and silent sites (synonymous sites and non-coding region) than for non-synonymous sites (Table 2). In addition, nucleotide variation and diversity were separately estimated (Table 3) by grouping the accessions into different phenotypic classes (muscat, neutral, aromatic, neutral Muscats and muscat-like aromatic mutants). The muscat class had a lower frequency of polymorphic sites (one every 62 bp) than the neutral class (one every 49 bp) and the dataset as a whole, but it was higher than the aromatic group (one every 76). The muscat-flavored accessions also have reduced nucleotide diversity (π = 0.0026, θ = 0.0029) compared with the neutral and aromatic classes and with the dataset as a whole.

**Table 2:** Overall polymorphisms in the *VvDXS *gene

All			Synonymous			Non-synonymous			Synonymous & non-coding		
			
S	π	θ	S	π	θ	S	π	θ	S	π	θ
102	0.003	0.0034	15	0.0056	0.0046	10	0.0012	0.001	92	0.0042	0.0046

S = segregating sites, π = nucleotide diversity per site, θ = Watterson's estimator

**Table 3:** Comparison of *VvDXS *nucleotide diversity in different phenotypic classes

Parameters	Overall (coding/non-coding)	Muscat (coding/non-coding)	Neutral (coding/non-coding)	Aromatic (coding/non-coding)	Neutral Muscats (coding/non-coding)	Muscat-like aromatic mutants (coding/non-coding)
Varieties	148	72	48	20	5	3

Number of polymorphic sites	102 (25/77)	77 (22/55)	99 (22/77)	64 (22/42)	48 (12/37)	26 (10/16)

Frequency of polymorphic sites	1 per 47 bp (1 per 86 bp/1 per 35 bp)	1 per 62 bp (1 per 98 bp/1 per 47 bp)	1 per 49 bp (1 per 98 bp/1 per 35 bp)	1 per 76 bp (1 per 98 bp/1 per 64 bp)	1 per 100 bp (1 per 179 bp/1 per 73 bp)	1 per 191 bp (1 per 215 bp/1 per 176 bp)

Synonymous changes	15	14	15	14	8	6

Non-synonymous changes	10	8	7	8	4	4

Synonymous vs non-synonymous mutations	1.5:1	1.75:1	2:1	1:75:1	2:1	1.5:1

Mean nucleotide diversity (π/θ)	0.0031/0.0034	0.0026/0.0029	0.0035/0.0040	0.0032/0.0034	0.0034/0.0036	0.0018/0.0023

*C*	10.8	1.999	16.605	9.9	nd	nd

Mean Tajima D	-0.19	-0.35	-0.42	0.67	nd	nd

Fu and Li's D*	0.91	1.58*	1.15	1.30	nd	nd

π = nucleotide diversity per site, θ = Watterson's estimator; nd = not determined; *C *= recombination parameters * = this value is significantly (P < 0.05) different from its neutral expectation based on the critical values obtained after coalescent simulations, polymorphic sites include SNPs and INDELs.

### In silico analysis of VvDXS protein and prediction of tolerability of amino acid exchanges

The prediction of tolerability of amino acid exchanges was evaluated for all ten non-synonymous mutation detected, and four were predicted to alter protein function (SIFT score < 0.05) by affecting either amino acid R-chain charge or amino acid polarity (Table 4). Among non-neutral mutations, S272P and R306C were found in Chardonnay musqué clone 44-60 Dijon and Gewürztraminer respectively whereas K284N was detected for 75 genotypes and S601F in 6 varieties. An additional non-synonymous change H11Y was found in VvDXS protein predicted from the cDNA of Moscato Bianco. This amino acid change was due to a polymorphism in the first 35 bp of the 5' coding region (at site 3764774 bp in NC_012011). There were too many missing data for this SNP, thus H11Y was not considered for the statistical analysis and it was not included in the total number of non-synonymous mutations. Anyway, it was predicted by SIFT as a tolerated mutation (SIFT score 0.50).

**Table 4:** Characteristics and in silico prediction of non-neutral non-synonymous changes

Sites (bp) NC_012011	SNP	Amino acid change	Amino acid substitutions characteristics	SIFT prediction (score)
3763971	SNP 772 (G/A)	T131A	polar neutral/non-polar neutral	tolerated (1.00)
3762959	SNP 1784 (T/C)	S272P	polar neutral/non-polar neutral	affect protein function (0.00)
3762921	SNP 1822 (G/T)	K284N	polar basic/polar neutral	affect protein function (0.00)
3762761	SNP 1982 (C/T)	R306C	polar basic/slightly polar neutral	affect protein function (0.00)
3760773	SNP 3970 (G/A)	T514A	polar neutral/non-polar neutral	tolerated (0.85)
3760635	SNP 4108 (G/A)	V560I	non-polar neutral	tolerated (0.11)
3760608	SNP 4135 (A/G)	I569V	non-polar neutral	tolerated (0.40)
3760485	SNP 4258 (G/A)	R584K	polar basic	tolerated (0.31)
3760434	SNP 4309 (C/T)	S601F	polar neutral/non-polar neutral	affect protein function (0.01)
3759957	SNP 4786 (C/T)	L716S	non-polar neutral/polar neutral	low confidence prediction

SNPs are named and scored according to their position on *VvDXS *ORF of *V. vinifera *PN40024; sites are referenced to the nucleotide positions relative to locus NC_012011. Low confidence prediction means there were not enough comparisons at that site.

### *VvDXS *intragenic LD estimation and haplotype structure detection

**Figure 2:**
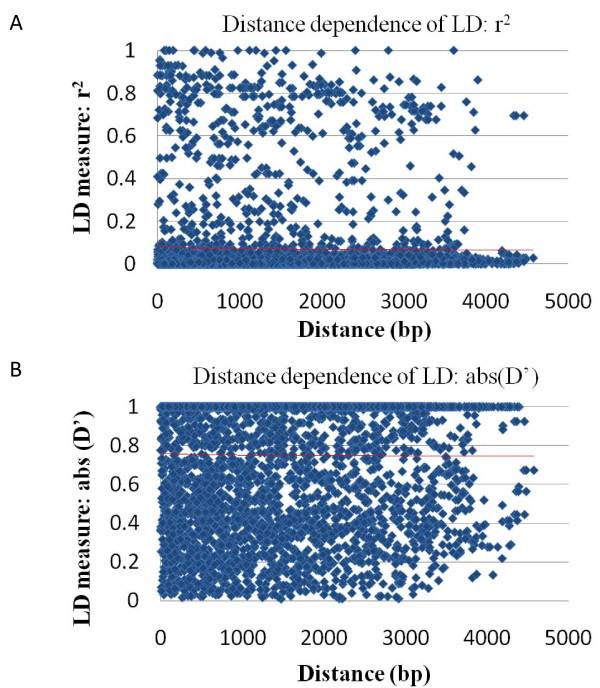
**Composite plot of linkage disequilibrium as a function of distance**. Two measures of linkage disequilibrium, r^2 ^(A) and absolute value of D' (B), are shown as a function of distance for *VvDXS*. LD values between all pairs of SNPs were plotted. Logarithmic trend line is included in both plots.

**Figure 3:**
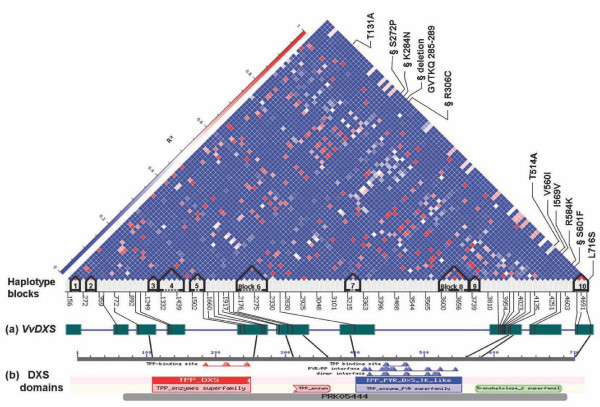
**LD plot, haplotype blocks and V*vDXS *gene structure**. The upper portion of the figure shows the LD plot based on r^2 ^values and the haplotype structure (ten blocks) for the 102 polymorphic sites identified in *VvDXS*. Non-synonymous mutations are located in the LD plot. All the polymorphisms detected in coding regions are positioned on (a) *VvDXS *gene structure (exons = boxes) and (b) the functional domains of DXS protein (conserved domain database, CDD http://www.ncbi.nlm.nih.gov/Structure/cdd/cdd.shtml). § = Non-neutral mutations predicted by SIFT.

Intragenic LD was estimated by calculating the square of the correlation coefficient of allele frequencies (r^2^) and the absolute value of D' for all pairs of polymorphic sites (Figure 2). R^2 ^and the absolute value of D' are both accepted measures for analysis of the distance dependence of LD. The mean r^2 ^for all 5022 pairwise comparisons was 0.0717 and the median was 0.0067, thus no intragenic LD measuring r^2 ^was observed, although several sites were still closely linked (r^2 ^> 0.6) over long distances (> 4500 bp). Instead, a significant gene-wide LD was found by evaluating absolute D' (mean absolute D' = 0.75 and median absolute D' = 1). The LD plot of r^2 ^values for all pairs of polymorphic sites and the detected haplotype blocks in function of the *VvDXS *gene structure and of the conserved domain organization of the predicted amino acid sequence are shown in Figure 3. Ten haplotype blocks were deduced, blocks 1, 2, 3, 4, 7, 8 and 9 being located within intronic regions and block ten identified within a coding region. Haplotype blocks five and six are mainly located in introns but they also include exonic SNPs (SNP 1594 and SNP 2176 respectively).

### Association testing

To avoid false positive results due to population stratification of the subset, we tested for associations with the structured association procedures.

With our sample, the best population subdivision revealed by STRUCTURE was obtained for K = 2 sub-populations and the corresponding Q matrix was first tested as an independent variable. Population structure effect was significant (4.60E-17), so the Q matrix was then included as covariate for association analysis. All 102 polymorphic sites revealed in the study were tested. Using the logistic regression model, 3 out of the 102 tests yielded a significant result after Bonferroni correction (Holm-Bonferroni threshold value of P = 0.05 set to 4.90E-04) with P-values ranging from 1.79E-05 to 1.31E-10 (Table 5). A G/T SNP at gene position 1822 was found to be significantly associated with aromatic and muscat-flavored varieties (T1822 allele being associated to Muscat type) with a smaller P-value than a G/A SNP at position 4108 and a T/G SNP at position 4175. SNP 4108 and SNP 4175 are linked to SNP 1822, with r^2 ^= 0.38 and r^2 ^= 0.61 respectively. SNP 1822 causes an amino acid change from K to N in position 284, whereas SNP 4108 causes a change from V to I in position 560 and SNP 4175 being synonymous.

**Table 5:** Significant sites identified with a structured association analysis

Sites (bp) NC_012011	Markers	Non-synonymous change	P-value covariate (Q matrix) Bonferroni adjusted	P-value Markers - Muscat flavor Bonferroni adjusted
3762921	SNP 1822 (G/T)	K284N	2.11E-05	1.31E-10
3760568	SNP 4175 (T/G)		6.40E-09	4.45E-08
3760635	SNP 4108 (G/A)	V560I	1.55E-13	1.79E-05

SNPs are named and scored according to their position on *VvDXS *ORF of *V. vinifera *PN40024; sites are referenced to the nucleotide positions relative to locus NC_012011.

Holm-Bonferroni threshold value of P = 0.05 set to 4.90E-04.

**Figure 4:**
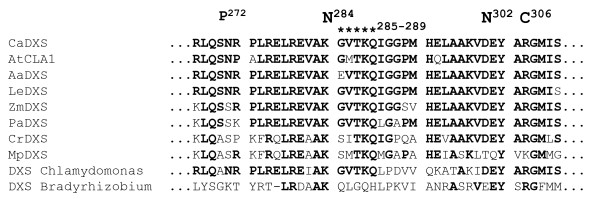
**Muscat and muscat-like aromatic mutants non-neutral substitutions**. The amino acid sequence of the region of the Muscats and muscat-like aromatic mutants non-neutral substitutions is shown for various plant and bacterial DXS. Identical amino acids are shown in bold type and the mutations are indicated above the relevant position. The amino acid residues that are substituted in Chardonnay Musqué clone 44-60 Dijon (S272P), Muscats (K284N), Gewürztraminer, Siegerrebe (R306C) and the putative deletion of GVTKQ(285-289) in Chasselas Musqué co-localize next to the mutations (D302N) responsible for the variegated phenotype of Arabidopsis *lvr111 *mutant. Full species names and GenBank accession numbers are as follows: Plant - AtCLA1 (*Arabidopsis thaliana*, NP_193291), CaDXS (*Capsicum annuum *, O78328), AaDXS (*Artemisia annua*, AF182286), LeDXS (*Lycopersicon esculentum*, AF143812), ZmDXS (*Zea mays*, ABP88134), PaDXS (*Picea abies*, ABS50518), CrDXS (*Catharanthus roseus*, CAA09804), MpDXS (*Mentha x piperita*, AAC33513); Bacteria - DXS *Chlamydomonas *(CAA07554), DXS *Bradyrhizobium *(YP 001204249).

In all 48 neutral varieties and in 4 out of 5 neutral varieties sharing a parentage with muscat genotypes, the allele carrying the mutated N at position 284 (Table 6) was not present. Regarding the 72 muscat-flavored genotypes, more than 95% of the accessions presented the mutation, 68 varieties in the heterozygous state and only one in the homozygous state. On the other hand, among aromatic individuals only 25% of the genotypes (5 out of 20 varieties) had the mutated allele N284, including one variety that presented the mutation in the homozygous state. Three muscat-like aromatic mutants (Gewürztraminer, Chardonnay musqué clone 44-60 Dijon and Chasselas musqué) and the aromatic cultivar Siegerrebe did not show the mutated allele N284 but instead exhibited unique heterozygous mutations. Gewürztraminer and Siegerrebe, which share a first degree parentage, both presented a change from R to C in position 306. Chardonnay musqué presented a non-synonymous change from S to P at site 272, whereas Chasselas musqué displayed a mutation in a splicing site responsible for a putative deletion of 5 amino acids from position 285 to position 289 (Table 7). All these non-neutral substitutions were located close to the Muscat K284N mutation (Figure 4).

**Table 6:** The relationship between SNP (G/T) 1822-based allele classification and aroma phenotypes

	Aroma phenotypes					
	
SNP (G/T) 1822-based alleles K - N mutation	Muscat (72)	Aromatic (20)	Neutral (48)	Neutral Muscats (5)	Muscat-like aromatic clones (3)	Tot (148)
Allele N284/allele K284	68	4	0	1	0	73
Allele N284/allele N284	1	1	0	0	0	2
Allele K284/allele K284	3	15	48	4	3	73

**Table 7:** Non-synonymous mutations and a putative amino acid deletion found in muscat-like aromatic mutants only

Sites (bp) NC_012011	SNP	Amino acid change	Muscat-like aromatic mutants
3762959	SNP 1784 (T/C)	S272P	Chardonnay musqué 44-60 Dijon
3762826	SNP 1917 (A/G)	Deletion GVTKQ 285-289	Chasselas musqué
3762761	SNP 1982 (C/T)	R306C	Gewürztraminer *

SNPs are named and scored according to their position on *VvDXS *ORF of *V. vinifera *PN40024. Sites are referenced to the nucleotide positions relative to locus NC_012011. * = the same mutation is also found in the aromatic cultivar Siegerrebe.

In order to identify polymorphisms associated with flavor intensity, tests were performed according to an ordinal linear regression model. However, they did not produce any significant results after Bonferroni correction. Thus, none of the tested polymorphic sites of *VvDXS *was found to be exclusively associated to either the high muscat-flavored groups or to the aromatic, low muscat and unstable phenotypes.

### Neutrality Tests and network analysis of reconstructed haplotypes

Ninety-six haplotypes were reconstructed taking into account all polymorphic sites detected.

#### Neutrality Tests

Tajima's D test (Table 3) did not reveal any significant departure from the neutral expectations and resulted in a slightly negative value for the dataset as a whole, and the muscat and neutral classes (D = -0.19, D = -0.35 and D = -0.42 respectively), and in a slightly positive value for the aromatic group (D = 0.67). Fu and Li's D* test (without an outgroup) yielded positive values for all the genotypic groups evaluated (Table 3) but the value was statistically significant (P < 0.05) only for the muscat class (D* = 1.58).

#### Network analysis of reconstructed haplotypes and haplogroups diversification

**Figure 5:**
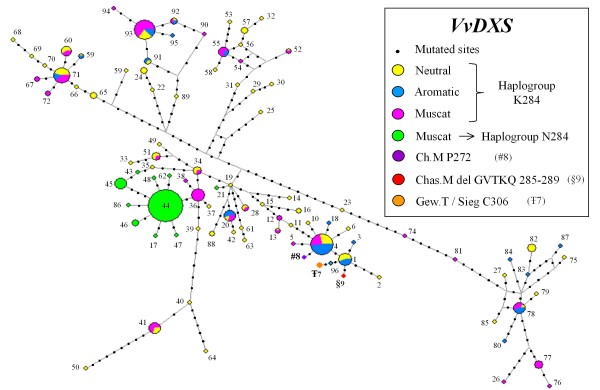
**Median Joining (MJ) networks derived from reconstructed DNA sequence haplotypes of *VvDXS***. Network analysis carried out on 96 haplotypes reconstructed from 102 polymorphic sites in *VvDXS *gene. Circles represent distinct haplotypes and are scaled to reflect their frequencies. The branches connect the haplotypes and indicate the mutational steps between them. Ch.M = Chardonnay musqué clone 44-60 Dijon, Chas.M = Chasselas musqué, Gew.T = Gewürztraminer, Sieg = Siegerrebe.

The MJ network analysis revealed a large diversity of the haplotypes with some major haplotypes shared across muscat-flavored, neutral and aromatic varieties. However, it also showed a major star-shaped cluster of *VvDXS *haplotypes carrying the mutation N284 (haplogroup N284) present only in Muscat genotypes (Figure 5). Haplotypes unique to Siegerrebe and Gewürztraminer (C306) and to muscat-like aromatic mutants Chasselas (del GVTKQ 285-289) and Chardonnay (P272) grouped into a distinct cluster together with two frequent haplotypes. These two common haplotypes correspond to the alleles also found in non-aromatic Chardonnay 130 and Chasselas respectively. Allele C306 and allele del GVTKQ 285-289 were linked to the Chasselas haplotype through single distinct mutations, whereas allele P272 was linked to the Chardonnay haplotypes. A reduced diversity in the number of segregating sites, in number of haplotypes as well as in nucleotide diversity was observed in haplogroup N284 (Additional file 2). Tajima D tests showed a rather negative and significant value (D = -1.71, P < 0.05) in the haplogroup N284 and a slightly positive but not significant one (D = 0.111) in the haplogroup K284. In addition, Fu and Li's D* test was negative and significant (P < 0.05) in the haplogroup N284 (D* = -2.71) and still positive and non significant (D* = 0.95) in the haplogroup K284.

## Discussion

The aim of the present study was to investigate the connection between the positional candidate gene *VvDXS *and muscat flavor in grapevine (*V. vinifera *L.) using an association genetics approach.

### Description and nucleotide diversity of the candidate gene *VvDXS*

*VvDXS *gene structure consists of ten exons and nine introns spread for a total of 4790 bp corresponding to the gene prediction LOC 100249323 on *V. vinifera *PN40024. A coding region of 2151 bp is predicted to encode for a DXS protein of 716 amino acids. The overall level of sequence polymorphism of *VvDXS *in grapevine is high and the overall SNP frequency is higher than the average frequency of polymorphisms (1 every 64 bp) described by Lijavetsky et al. [47] for 230 gene fragments in 10 grape genotypes. On the other hand, the overall SNP frequency observed here is slightly lower than the frequency described by Le Cunff et al. [42] (1 every 49 bp) for three genes in the G-92 core collection. Moreover, the ratio of synonymous to non-synonymous changes in *VvDXS *(1.5:1) is higher than the 1:1 reported by Ljiavetsky et al. [47]. Forty percent of the missense mutations were predicted to affect protein function, which is again higher than the 16% observed by Lijavetsky et al. [47]. When considering the subsets of muscat, neutral and aromatic accessions separately, the polymorphic site frequency and the mean nucleotide variability were higher in the neutral group than in the muscat and aromatic groups. This is not surprising, as 45 out of 48 neutral genotypes belong to the G-48 core collection, which was designed to represent a huge percentage of the genetic variability in a grapevine collection [42], while muscat types share common ancestry to a certain extend.

### *VvDXS *intragenic LD estimation and haplotype structure detection

Pairwise LD was evaluated by calculating r^2 ^and absolute D' parameters for SNP loci within the *VvDXS *sequence. The r^2 ^calculation revealed an absence of overall intragenic LD, even though several sites were in significant LD over long distances. On the other hand, absolute D' showed significant gene-wide LD. These contradictory results may be due to the large number of minor haplotypes in *VvDXS *caused by mutations rather than recombination events. Indeed, somatic mutations combined with vegetative propagation may have played a major role in increasing the genetic diversity in cultivated grapevine [51]. However, intragenic and short-range LD in grapevine have been shown to decay rapidly. This et al. [49] reported in *VvMybA1 *gene an r^2 ^value of 0.2 along 700 nucleotides and then a rapid decay. Lijavetsky et al. [47] observed in more than 200 gene fragments a decay of absolute D' and r^2 ^between 100 and 200 bp and, consistent with these data, Myles et al. [48] recently found low levels of LD (r^2 ^< 0.2) even at short physical distances with massive genotyping. On the other hand, significant long-range LD was reported by Barnaud et al. [46] in cultivated grapevine using SSR markers, a discrepancy that has been observed in other species such as maize [45] and humans [52]. However, Barnaud et al. [53] more recently observed a rapid decay of long-range LD in the French wild grapevine. Most of the haplotype blocks identified in the present study are located within introns, whereas only one haplotype block 10 exclusively covers a coding region. The exonic SNP 1594 and SNP 2176 are located within the pyrophosphate (TPP-PP) and pyrimidine (TPP-PYR) protein domains of DXS, these being involved in the binding of Thiamine pyrophosphate (TPP). These domains are conserved among DXS proteins and are very similar in all the TPP cofactor-dependent proteins (i.e. transketolase, pyruvate oxidase, pyruvate decarboxylase, etc). The functional significance of these regions may explain the presence of two major haplotype blocks (five and six) which show several intronic polymorphisms in LD with the exonic ones.

### Association testing

Allelic variation in *VvDXS *was associated with Muscat flavor in cultivated grapevines sampled to maximize flavor diversity. More aromatic (muscat or other special flavored) accessions than non-aromatic individuals were evaluated in this analysis. In a case-control study, case and control groups are normally equally represented. In this study, controls were selected in order to retain a high percentage of the microsatellite diversity of a large grapevine germplasm collection. The wide genetic variability within the controls and the presence of 5 accessions sharing a parentage with muscat genotypes but not aromatic (asymptomatic), allowed us to overcome the unequal case-control ratio. Moreover, correction for the genetic structure of the sample increases protection against spurious associations compared to the Chi-squared statistic that is normally applied in simple case-control studies. According to the optimal K, all the accessions in this study divided into two genetically distinct pools. A significant population structure effect was observed on trait variation probably as a consequence of the over-representation of Muscat genotypes. Modern Muscats share a strong a family structure and are thought to descend from two very ancient grapevine cultivars, Moscato Bianco and Muscat of Alexandria [2]. The observed population subdivision therefore reflects a possible divergent selection for muscat flavor [1,51] that took place in the eastern Mediterranean basins [54]. When testing aromatic and muscat-flavored genotypes vs non-aromatic accessions in structured association, three SNPs (SNP 1822, SNP 4108 and SNP 4175) were found to be significant after Bonferroni correction. Nonetheless, given the high LD level among the significant SNPs, and the higher P-values of SNP 4108 and SNP 4175 we may also conjecture that their association with muscat flavor is due purely to linkage with SNP 1822. Moreover, these three SNPs do not fall into any haplotypic block deduced within the *VvDXS *gene. No significant association was detected to distinguish between aromatic and muscat-flavored fruited varieties, nor to explain flavor intensity variation within the aromatic and muscat groups. Therefore, none of the tested polymorphic sites of *VvDXS *can explain either a quantitative or qualitative effect responsible for the aromatic to muscat flavor transition. The SNP 1822 causes a non-synonymous amino acid change. Lysin at position 284 is replaced by an Asparagine in over 95 percent of the muscat-flavored genotypes under study. Three Muscat-flavored accessions did not carry that mutation; it is therefore likely that there are other muscat or aromatic mutations in the cultivated grapevine which may be far rarer or may not have spread within *V. vinifera*. The muscat accessions identified here that do not contain the N284 non-synonymous change in the *VvDXS *coding region may well contain mutations in other candidate genes, such as *DXR*, *IDS *and *HDR*, which may also contribute to the metabolic flux through the MEP pathway [55-57]. The hypothesis that there exist other rare mutations leading to aromatic or muscat-flavored phenotypes is reinforced by the analysis of the muscat-like aromatic mutants and the heterogeneous aromatic group. Interestingly, as in the muscat-like aromatic mutants of Chardonnay, Chasselas and Savagnin rosé, unique, distinct, non-neutral mutations have taken place independently in the coding region of *VvDXS *near to the muscat mutation N284. The muscat-like aromatic mutant of Savagnin rosé (Gewürztraminer) and the aromatic cultivar Siegerrebe, for which a direct parent-offspring relationship has been postulated, show the same non-synonymous change, confirming that this mutation was inherited together with the characteristic flavor. Moreover, Chardonnay musqué clone 44-60 Dijon has a single heterozygous mutation that is absent in the neutral clone Chardonnay 130. The low presence of N284 alleles in the aromatic group needs to be carefully evaluated due to the heterogeneous nature of the accessions. Indeed, varieties showing fruity or floral flavor other than the distinct muscat aroma may produce different kinds of free aromatic compounds. Moreover, where genotypes exhibit a very slight muscat flavor, this is often hardly perceived and they are more generally classified as aromatic. A group of five aromatic accessions (Albalonga, Aromriesling, Bouquettraube, Bouquet Sylvaner, Jo Rizling) sharing parentage with Rhine Riesling, did not carry the mutation N284. The characteristic aroma of these accessions mainly depends on C_13 _norisoprenoid accumulation in the berry skin rather than on monoterpenoids. These genotypes accumulate monoterpenoids in higher levels compared with the non-aromatic varieties, but in significantly lower amounts compared with Muscats [58]. Even though monoterpenoids and C_13_-norisoprenoids share a common precursor, isopentenyl diphosphate (IPP), it is reasonable to assume that the divergent pathways giving rise to their production are under different genetic controls. In all 48 neutral varieties of core G-48 and in 4 out of 5 neutral varieties sharing parentage with muscat genotypes, the N284 mutation was absent. Only Muscat Lierval presented the missense change, even though it was classified as non-aromatic. This is an exception that should be further investigated by carrying out a quantification of monoterpenoid content in order to confirm the phenotypic evaluation obtained by tasting. In any case, quantitative estimates of monoterpenoid concentrations for all Muscats and aromatic genotypes would help to increase the power and accuracy of the association test. This is particularly necessary when testing polymorphisms that may explain minor effects in muscat flavor determination compared to the N284 mutation.

### Neutrality Tests and network analysis of reconstructed haplotypes

#### Neutrality Test

Statistical tests of neutrality on the basis of the site frequency spectrum are known to be confounded by demographic processes [59,60]. Therefore, these results need to be carefully managed also considering the size and the genetic structure of the sample studied. Analysis of site frequency distributions using the Tajima D test did not reveal any significant departure from neutrality in the dataset as a whole and in the subsets of muscat, neutral and aromatic genotypes. Similarly, the null hypothesis of evolutionary neutrality was not rejected by Fu and Li's D* test (without an outgroup) except in the case of the muscat class. A significantly positive Fu and Li's D* describes an excess of heterozygosity in the muscat group.

#### Network analysis of reconstructed haplotypes and haplogroups diversification

There is little sequence diversity within the muscat-flavored allele of *VvDXS *containing the N284 mutation. This narrow genetic variability is confirmed by the negative and significant values of Tajima D and Fu and Li's D* (Additional file 2) detected by grouping the haplotypes carrying the N284 mutation. This observation and the presence of a star-shaped cluster observed in the MJ analysis suggest that the muscat-flavored allele most likely arose only once quite recently, and it underwent a strong selective pressure or most likely an exponential growth due to intense breeding practice. In the opposite, Tajima D and Fu and Li's D* are both positive but not statistically significant for the haplogroup K284, which grouped the remaining haplotypes. This result assess that there are no evidence of a human-driven selection on *VvDXS *alleles that do not carry the K284N mutation. In addition, the MJ analysis shows some major haplotypes among the haplogrouop K284 shared by muscat-flavored, neutral and aromatic accessions. These observations suggest a common pool of neutral varieties used in the breeding practices of both Muscats and Non-Muscats grapevines. Muscat genotypes share a strong genetic family structure while displaying considerable phenotypic variability for traits such as berry color, flowering and ripening time. It is also well known that Muscats have been extensively used by grape breeders to obtain several popular crosses for table grapes and for wine. The excess of heterozygosity detected in the muscat group and the narrow genetic variability observed in the N284 haplogroup may reflect the breeding history of the Muscat family. We suggest an initial selection for muscat flavor, with subsequent crosses between muscat and neutral genotypes. Individuals displaying muscat aroma and the desired phenotypic characteristics inherited from the neutral parent were then selected and vegetatively propagated by grafting. This way, the N284 mutation was selected and bred in its heterozygous state in the majority of the muscat-flavored varieties in existence today. In any case, we cannot yet exclude the possibility that homozygosity of the N284 mutation may affect grape fitness by reducing flower fecundity and seed fertility. The MJ Network also shows that the mutated alleles P272 (Chardonnay musqué 44-60 Dijon), C306 (Gewürztraminer and Siegerrebe) and del GVTKQ 285-289 (Chasselas musqué) arose independently from single mutations of non-aromatic Chardonnay 130 and Chasselas haplotypes.

### Putative functional effect of the polymorphisms

The crucial role of the DXS protein has been studied in bacteria and in plants [15,22] and its sequence is highly conserved, although it also shows a weak sequence homology with transketolase (TK) [61-63]. Residues 267-312 of *V. vinifera *VvDXS correspond to a segment located near the active site in domain I of *Deinococcus radiodurans*. Co-located in this region are the non-neutral mutations found in Muscats (K284N), Gewürztraminer and Siegerrebe (R306C), and Chardonnay musqué (S272P), as well as the 5 amino acid deletion found in Chasselas musqué (285-289) caused by a point mutation in a splicing site. In the case of Muscats, Gewürztraminer and Siegerrebe, the substitutions alter the amino acid R-chain charge with positively charged amino acids (Lysin and Arginine) being replaced by neutral amino acids (Asparagine and Cystein). Lysin at position 284 is highly conserved in DXS in plants as well as in algae and bacteria (Figure 3), whereas Serine at position 272 and Arginine at position 306 are not so highly conserved. Interestingly, Arabidopsis *lvr111 *mutant [24] presents a D306N change in 1-deoxy-D-xylulose 5-phosphate synthase (corresponding to D302 in *V. vinifera *VvDXS) which is located close to the non-neutral changes reported in our study. This mutation in DXS causes a reduction in chlorophyll accumulation, so that the *lvr111 *mutant shows a semi-dominant variegated phenotype under normal growth conditions. These residues do not correspond to the conserved DRAG sequence of DXS [64] nor to the other conserved positions identified in DXS or TKs [65-69]. This may presumably explain the non-lethal effect of amino acid replacement in this protein region. Some recent reports have also demonstrated that DXS is regulated at post-transcriptional levels [19-21], so we should not exclude the possibility that these amino acid substitutions affect protein turnover.

## Conclusions

Our results confirm the role of *VvDXS *in determining muscat flavor in grapevine. For the first time, to our knowledge, an SNP in the coding region of *VvDXS *has been suggested as the causal "gain of function" mutation. Besides a clear genetic separation between muscat-flavored and neutral varieties, our results highlight *VvDXS *as an important human-selected locus. We suggest that *VvDXS *underwent a strong selection in the group of Muscats, due to specific and intense breeding practices during grapevine domestication and post-domestication. In addition, by analyzing the nucleotide sequence of *VvDXS *we were able to identify independent mutations in the same region of the gene giving rise to muscat-like aromatic mutants from neutral clones of Chardonnay, Chasselas and Savagnin rosé. This discovery highlights the existence of distinct mutations unique to the muscat-like aromatic mutants under study, as opposed to the SNP found in Muscats. Further studies are required to assess the functional effect of these putative causal mutations. Nevertheless, these polymorphisms may be immediately applied in marker-assisted selection (MAS) for rapid screening of seedlings with the potential to express the muscat flavor.

## Methods

### Plant material and phenotypic data

The association population consisted of one hundred and forty-eight grapevine (*V. vinifera *L.) accessions held by the French national grapevine germplasm collection at "Domaine de Vassal", France [46]. This population includes 47 genotypes of the "G-48 core collection" [42], which encompasses more than 80% of the microsatellite diversity found within this species. Seventy-two muscat-flavored and twenty aromatic (other special flavor) accessions were sampled to maximize flavor diversity. Forty-eight neutral varieties and five non-aromatic accessions sharing parentage with Muscat varieties were also included. In addition, three muscat-like aromatic mutants of Savagnin rosé (Gewürztraminer), Chardonnay (Chardonnay musqué clone 44-60 Dijon) and Chasselas (Chasselas musqué), were investigated. The complete list is reported in Additional file 3. Muscat flavor was scored in different years by trained tasters who described the accessions as either non-aromatic, aromatic or muscat. Tasters were trained by tasting several berries of different clusters of example samples as indicated by OIV Descriptors for Grapevine - OIV code number 236- [70]. Grape aroma was thus scored according to a 3-point scale: 0 = non-aromatic, 1 = muscat (light and high muscat-flavored), 2 = other special flavor (light and high aromatic). Aromatic and muscat-flavored accessions that were perceived as non-aromatic by the majority of the tasters in at least one season were considered respectively as aromatic unstable and muscat unstable. The average score was used in further analyses.

### Validation of the candidate gene *VvDXS *expression into Muscat genetic background

#### RNA extraction and cDNA synthesis

The skin and pulp of 40 berries of *V. vinifera *'Moscato Bianco', sampled from pre-véraison to over-ripening, were separately frozen in liquid nitrogen and then stored at -80°C for RNA extraction. Total RNA was extracted in triplicate from pericarp tissue of each sample using the SIGMA Spectrum™ Plant Total RNA Kit. RNA concentration and 260/280 nm ratio were determined before and after DNase I digestion (Invitrogen) with a spectrophotometer and RNA integrity was checked by electrophoresis on 1.5% agarose gels in 0.5 X TAE (90 mM Tris-acetate, 2 mM EDTA). First strand cDNA was synthesized from total RNA using Superscript™ III Reverse Transcriptase (Invitrogen) according to the manufacturer's instructions.

#### Cloning Moscato Bianco full-ORF *VvDXS *cDNA alleles

A cDNA pool equally representing each sampled ripening stage was then used as a template for full-ORF *VvDXS *cDNA amplification. The full-ORF *VvDXS *cDNA was amplified by PCR using high fidelity Phusion polymerase (Finnzymes) with the forward primer cVvDXS-fw 5-CACCATGGCTCTCTGTACG-3 and the reverse primer cVvDXS-rw 5-CTATGACATGATCTCCAGGGC-3, corresponding to the start and the end of the coding region of *VvDXS*. Primer cVvDXS-fw contains the sequence CACC at the 5' end of the primer to permit directional cloning in pENTR/D-TOPO (Invitrogen). PCR conditions were: 98°C for 30 s followed by 35 cycles of 98°C for 10 s, 65.8°C for 30 s, 72°C for 1 min, with a final elongation step of 72°C for 10 min. PCR products were purified from agarose gel using PureLinkTM Quick Gel Extraction and the PCR Purification Combo Kit (Invitrogen, California) according to manufacturer's instructions. Fragments were subcloned into vector pENTR/D-TOPO (pENTR™ Directional-TOPO cloning kit, Invitrogen, Califonia) and *E. coli *One Shot TOP10 was employed as the host strain for gene manipulation. Forty-eight clones were randomly picked and the allele-specific ones were identified by colony PCR screening. *Sty*I digestion (3 hs at 37°C followed by 5 min at 65°C, 1 unit, Fermentas) was used to distinguish the two Moscato Bianco *VvDXS *alleles, A and B. PCR conditions were the same as those described for genomic resequencing of *VvDXS *with the forward primer M13fw and the reverse primer ex_dxs_2rw sequences listed in Additional file 4. Clones containing the A and B allele were renamed pENTR/D-TOPO:*VvDXS*A and pENTR/D-TOPO:*VvDXS*B respectively and selected for plasmid DNA extraction with the Genelute Plasmid Mini-prep Kit (Sigma) according to the manufacturer's instructions. Purified plasmid DNA of twelve out of the 48 allele-specific clones was resequenced using primers listed in Additional file 4, as described above.

### Description and nucleotide diversity of the candidate gene *VvDXS*

#### *VvDXS *amplification and resequencing

Genomic DNA was extracted from 20 mg of freeze-dried leaf material using the DNeasy kit (Qiagen, Hilden) according to the manufacturer's protocol. The *VvDXS *gene was amplified and directly sequenced in 148 accessions. Gene-specific primers were designed and synthesized based on the genomic sequence of *V. vinifera *PN40024 deposited in the NCBI genome chromosome database under accession number NC_012011. A total of 4790 bp of the *VvDXS *locus, from the initial ATG start codon to the TAG stop codon, was resequenced corresponding to base pair numbers 3759954-3764743 of NC_012011. Both strands of eight partially overlapping amplicons were sequenced and assembled in a contiguous sequence. Primers used to amplify PCR fragments were also employed for the resequencing and are listed in Additional file 5. The polymerase chain reaction (PCR) mixture (12.5 μl) contained 5-10 ng of genomic DNA, 1.25 μl of 10× PCR buffer (QIAGENE, Valencia, CA, USA; 1.5 mM of MgCl_2_), 40 μM of each dNTP, 0.6 μM of each primer and 0.5 unit of HotStarTaq polymerase (QIAGENE). Amplification was carried out using a GeneAmp PCR System 9700 (Perkin-Elmer, Norwalk, CT, USA) and a touchdown protocol [71]. Thermocycling consisted of an initial denaturation of the template DNA at 95°C for 15 min, followed by 11 cycles of 95°C for 45 s, 62°C (touchdown step from 62°C to 57°C) for 45 s and 72°C for 1 min, and another 24 cycles of 95°C for 45 s, 57°C for 45 s and 72°C for 1 min, with a final extension of 10 min at 72°C. Reaction products were analyzed in 1.5% agarose gels buffered in 0.5 X TBE (90 mM Tris-borate, 2 mM EDTA) and visualized by UV-light after staining with ethidium bromide (1 μg/ml). Two to four nanograms of amplified DNA were employed for every 100 bp to be sequenced in both directions. PCR products were purified with ExoSapIT (Amersham Pharmacia Biotech, Uppsala, Sweden) and sequenced with the Big Dye^® ^Terminator v 3.1 Cycle Sequencing Kit (Applied Biosystems) in a GeneAmp PCR System 9700 according to the manufacturer's instructions. After precipitation, the sequencing products were mixed with 15 μl of HiDi™ formamide and subjected to capillary electrophoresis in an ABI PRISM 3130xl Genetic Analyzer (Applied Biosystems). Sequences were processed with the Sequencing analysis v 3.7 software (Applied Biosystems) then assembled and manually inspected with a STADEN package ver 1.5.3.

#### Nucleotide polymorphisms and diversity in the candidate gene *VvDXS*

Based on the SNPs, INDELs and *VvDXS *cDNA sequence detected, the nature and frequency of polymorphisms were defined using the DnaSP program [72]http://www.ub.es/dnasp. Nucleotide diversity was evaluated with the parameter π [73], which is the average number of nucleotide differences per site between two sequences. The neutral mutation parameter θ [74] was calculated from the total number of mutations.

#### In silico analysis of VvDXS protein and prediction of tolerability of amino acid exchanges

Predicted VvDXS proteins were aligned using MEGA 4 software [75]. Prediction of tolerability of amino acid exchange at all positions was calculated using the SIFT software [76]http://blocks.fhcrc.org/sift/SIFT.html.

#### Linkage disequilibrium analysis and haplotype structure detection

Linkage disequilibrium measures r^2 ^[77] and absolute D' [78] were calculated using the DnaSP and TASSEL software ver. 2.1 [79]http://www.maizegenetics.net/tassel. Fisher's exact test was applied to calculate the significance of pairwise LD when using DnaSP, while 1000 permutations were performed using TASSEL ver. 2.1. Haplotype blocks, detected using the method described by Gabriel et al. [80] and the LD plot of r^2 ^values were evaluated using the Helixtree software package (Golden Helix).

### Association genetics

#### Population structure

To avoid false positive associations due to genetic stratification of the population under study, all 148 accessions were genotyped at 20 genome-wide microsatellite (SSRs) loci [81]. SSR data were used to infer the population structure using the STRUCTURE 2.1 software [82,83]. This software applies a Bayesian clustering approach to identify sub-populations and assign individuals to them while simultaneously estimating the allele frequencies in the populations. STRUCTURE produces a Q-matrix that lists the estimated membership coefficients for each individual in each cluster. The ADMIXTURE model was applied and segregation of alleles was assumed to be independent. A burn-in length of 1,000,000 followed by 1,500,000 iterations was used to estimate the Q-matrix for each population from one to ten [46]. Ln Pr(X/K) was calculated, where Pr denotes posterior probability, X denotes genotypes of the sampled individuals, and K denotes the assumed population number. The optimal sub-population model, i.e. the K with the highest posterior probability, was selected using Evanno's correction [84].

#### Association statistical test

The estimated Q-matrix was used in the subsequent association analysis which was carried out by logistic regression in the TASSEL ver. 1.4 software [79]http://www.maizegenetics.net/tassel. The logistic regression model was also fitted in R using the General Linear Model (GLM) function adapted to binary data (nonaromatic = 0; aromatic and muscat = 1) and implemented in Rcmdr, a platform-independent menu interface to R.

An ordinal linear regression analysis was then carried out using Rcmdr to identify polymorphisms associated with flavor intensity (with phenotypic data scored on a 3-point ordinal scale). Three almost equally-represented ordinal classes were defined (1 = neutral, 2 = aromatic/light muscat/muscat unstable, 3 = high muscat) and polymorphisms were tested incorporating the Q-matrix as covariate to class ordinal variation (class 1 to class 2 and class 2 to class 3). An ANOVA test was used to check for type II errors occurring when a false null hypothesis is not rejected.

### Neutrality Tests and network analysis of reconstructed haplotypes

#### Neutrality Tests

Tajima's D test and Fu and Li's D* test (without an outgroup) implemented in DnaSP were used to estimate neutrality of the SNP polymorphisms, taking the dataset as a whole and the muscat, neutral and aromatic classes into consideration separately. Neutral Muscats and muscat-like aromatic mutants were not tested separately because of the low number of individuals. Critical values for the above tests were calculated by coalescent simulations. As recombination tends to make these tests conservative [59,60], coalescent simulations were run to account for the level of recombination *C *[85] observed in the *VvDXS *sequences in each class. The 95% confidence intervals of the neutral distributions were calculated using 10,000 coalescent simulations in DnaSP, and statistical significance was inferred where the observed value lay outside these (p < 0.05).

#### Network analysis of reconstructed haplotypes

Due to the heterozygous nature of the sequence, haplotypes of the *VvDXS *gene were reconstructed using the Partition-Ligation expectation Maximization (PLEM) algorithm described in Qin et al. [86] and implemented in PHASE v2.1 [87]. A 200 burn-in with 200 iterations in total and a thinning interval of 1 was repeated 10 times until convergence was validated. Median-Joining (MJ) Networks [88] were constructed with the Network 4.1.1.2 program (Fluxus Technology Ltd, Clare, Suffolk, UK). Haplogroups N284 and K284 were defined based on the presence or absence of the polymorphism SNP (G/T) 1822 that causes the amino acid substitution K284N associated to muscat-flavored varieties Nucleotide diversity and tests of neutrality were performed as described previously by treating these haplogroups separately

## List of abbreviations

bp: base pairs; *chs5*: chilling sensitive 5; *cla1-1*: altered chloroplast 1-1; *DXR*: 1-deoxy-D-xylulose-5-phosphate reductoisomerase; *HDR*: 1-hydroxy-2-methyl-2-(E)-butenyl 4-diphosphate reductase; *IDS*: isopentenyl diphosphate/dimethylallyl diphosphate synthase; INDEL: INsertion-DELetion polymorphism; *lvr111*: lovastatin resistant 111; ORF: Open Reading Frame; QTL: Quantitative Trait Locus; SNP:Single Nucleotide Polymorphism; SSR:Simple Sequence Repeat.

## Authors' contributions

FE participated in the design of the study, carried out the genomic DNA extraction and the full-ORF *VvDXS *cDNA cloning, performed sequencing data analysis, carried out the association tests and network analysis and drafted the manuscript. JB carried out the sampling of Moscato Bianco berries, performed RNA extraction and the cDNA synthesis, provided support in the bioinformatic analysis, in the association study and contributed to the manuscript writing. LC contributed to defining the genotypes of the association populations and to writing the manuscript. LLC contributed to performing the association study and network analysis and contributed to critically reviewing the manuscript. JMB provided basic plant material and the phenotypic data and contributed to reviewing the manuscript. PT contributed to the design of the study and critically contributed to the discussion of the results and to reviewing the manuscript. MSG conceptualized the project and contributed to the discussion of the results and to reviewing the manuscript. All authors read and approved the final manuscript.

## Supplementary Material

Additional file 1 **List of the polymorphic sites detected and evaluated in the SA analysis**. SNPs and INDELs are named and scored according to their position on *VvDXS *ORF of *V. vinifera *PN40024; sites are referenced to the nucleotide positions relative to locus NC_012011. Click here for file

Additional file 2 **Comparison of *VvDXS *nucleotide diversity in haplogroups N284 and K284**. Haplotypes are divided into two haplogroups (N284 and K284) based on the SNP (G/T) 1822 responsible for the K284N substitution. π = nucleotide diversity per site, θ = Watterson's estimator; *C *= recombination parameter; * = this value is significantly (P < 0.05) different from its neutral expectation based on the critical values obtained after coalescent simulations. Click here for file

Additional file 3 **List of the *V. vinifera *L. accessions analyzed in this study**. 1 = neutral; 2 = aromatic; 3 = muscat flavor; § = unstable or slightly flavored; # = muscat-like aromatic mutants; * = neutral Muscats; Ŧ = FEM-IASMA accession; K284N = replacement of a Lysine with an Asparagine at site 284 caused by the SNP 1822. Click here for file

Additional file 4 **List of primers used in colony PCR and cloned cDNA *VvDXS *allele sequencing**. Click here for file

Additional file 5 **List of primers used for *VvDXS *genomic sequencing**. Click here for file
