# Supplementary material for: A candidate gene association study on muscat flavor in grapevine (Vitis vinifera L.)
Source: BMC Plant Biol. 2010 Nov 9;10:241. doi: 10.1186/1471-2229-10-241 (PMC3095323; doi:10.1186/1471-2229-10-241)
Supplement: Additional file 1 — List of the polymorphic sites detected and evaluated in the SA analysis. SNPs and INDELs are named and scored according to their position on VvDXS ORF of V. vinifera PN40024; sites are referenced to the nucleotide positions relative to locus NC_012011. [file 1471-2229-10-241-S1.PDF]

Additional file 1.pdf

List of the polymorphic sites detected and evaluated in the SA analysis.

Number of polymorphic sites	Polymorphic sites position relative for locus NC_012011 (bp)	Polymorphic sites position on *VvDXS* ORF of *V. vinifera* PN40024 (predicted on the minus strand of locus NC_012011)	Nucleotide substitution	Insertion / deletion respect to the reference sequence
1	3764587	SNP 156	C/T	
2	3764492	SNP 251	A/G	
3	3764472	SNP 271	G/C	
4	3764471	SNP 272	T/G	
5	3764387	SNP 356	G/C	
6	3764386	INDEL 357	C	insertion
7	3764384	SNP 359	T/A	
8	3764188	SNP 555	G/T	
9	3764145	SNP 598	A/G	
10	3763971	SNP 772	G/A	
11	3763923	SNP 820	A/T	
12	3763864	SNP 879	A/G	
13	3763851	SNP 892	A/G	
14	3763755	SNP 988	A/G	
15	3763680	SNP 1063	A/T	
16	3763494	SNP 1249	A/G	
17	3763490	SNP 1253	T/C	
18	3763460	SNP 1283	T/C	
19	3763411	SNP 1332	A/G	
20	3763403	SNP 1340	T/C	
21	3763331	SNP 1412	A/G	
22	3763304	SNP 1439	A/G	
23	3763271	SNP 1472	A/G	
24	3763248	SNP 1495	C/T	
25	3763241	SNP 1502	A/G	
26	3763221	SNP 1522	C/T	
27	3763149	SNP 1594	C/T	
28	3763083	SNP 1660	T/G	
29	3762959	SNP 1784	T/C	
30	3762921	SNP 1822	G/T	
31	3762826	SNP 1917	A/G	
32	3762761	SNP 1982	C/T	
33	3762591	SNP 2152	C/T	
34	3762567	SNP 2176	C/T	
35	3762536	SNP 2207	A/G	
36	3762473	SNP 2270	C/T	
37	3762468	SNP 2275	C/G	
38	3762420	SNP 2323	A/G	

39	3762419	SNP 2324	A/G	
40	3762413	INDEL 2330	ACAGAAAGTAGAATTATTA AATTCAAAAATGGACAT	deletion
41	3762336	SNP 2407	G/T	
42	3762080	SNP 2663	A/G	
43	3761913	SNP 2830	C/T	
44	3761874	SNP 2869	C/T	
45	3761862	SNP 2881	A/G	
46	3761818	SNP 2925	A/G	
47	3761705	SNP 3038	C/T	
48	3761699	SNP 3044	A/G	
49	3761695	SNP 3048	A/G	
50	3761683	SNP 3060	C/T	
51	3761664	SNP 3079	C/G	
52	3761642	SNP 3101	A/G	
53	3761636	INDEL 3107	T	deletion
54	3761535	SNP 3208	C/T	
55	3761528	SNP 3215	A/T	
56	3761477	SNP 3266	A/C	
57	3761420	INDEL 3323	CT	insertion
58	3761380	SNP 3363	A/G	
59	3761358	SNP 3385	C/T	
60	3761357	SNP 3386	A/G	
61	3761347	SNP 3396	T/C	
62	3761325	SNP 3418	T/C	
63	3761303	INDEL 3440	A	insertion
64	3761255	SNP 3488	C/T	
65	3761223	SNP 3520	T/G	
66	3761202	SNP 3541	C/T	
67	3761199	SNP 3544	T/G	
68	3761196	SNP 3547	C/T	
69	3761192	SNP 3551	A/G	
70	3761178	SNP 3565	T/C	
71	3761166	SNP 3577	A/T	
72	3761152	SNP 3591	A/T	
73	3761143	SNP 3600	C/A	
74	3761113	SNP 3630	T/G	
75	3761088	SNP 3655	G/A	
76	3761087	SNP 3656	C/T	
77	3761081	SNP 3662	C/T	
78	3761057	SNP 3686	A/G	
79	3761004	SNP 3739	T/C	
80	3761003	INDEL 3740	T	deletion
81	3760950	SNP 3793	A/G	
82	3760933	SNP 3810	C/T	
83	3760932	SNP 3811	A/G	
84	3760804	SNP 3939	C/T	
85	3760789	SNP 3954	T/C	
86	3760774	SNP 3969	T/C	
87	3760773	SNP 3970	G/A	
88	3760720	SNP 4023	C/T	

89	3760687	SNP 4056	C/T	
90	3760635	SNP 4108	G/A	
91	3760608	SNP 4135	A/G	
92	3760568	SNP 4175	T/G	
93	3760545	SNP 4198	A/G	
94	3760492	SNP 4251	A/G	
95	3760485	SNP 4258	G/A	
96	3760434	SNP 4309	C/T	
97	3760160	SNP 4583	C/A	
98	3760140	SNP 4603	C/T	
99	3760134	INDEL 4609	A	insertion
100	3760079	SNP 4664	C/T	
101	3760052	SNP 4691	A/G	
102	3759957	SNP 4786	C/T	

SNPs and INDELs are named and scored according to their position on *VvDXS* ORF of *V. vinifera* PN40024; sites are referenced to the nucleotide positions relative to locus NC_012011.
