# Supplementary material for: A candidate gene association study on muscat flavor in grapevine (Vitis vinifera L.)
Source: BMC Plant Biol. 2010 Nov 9;10:241. doi: 10.1186/1471-2229-10-241 (PMC3095323; doi:10.1186/1471-2229-10-241)
Supplement: Additional file 2 — Comparison of VvDXS nucleotide diversity in haplogroups N284 and K284. Haplotypes are divided into two haplogroups (N284 and K284) based on the SNP (G/T) 1822 responsible for the K284N substitution. π = nucleotide diversity per site, θ = Watterson's estimator; C = recombination parameter; * = this value is significantly (P < 0.05) different from its neutral expectation based on the critical values obtained after coalescent simulations. [file 1471-2229-10-241-S2.PDF]

Additional file 2.pdf

Comparison of *VvDXS* nucleotide diversity in haplogroups N284 and K284.

	VvDXS haplogroups	
	N284	K284
Segregating sites	12	101
Number of haplotypes	78	219
Mean nucleotide diversity (π / θ)	0.00019 / 0.00051	0.0037 / 0.0035
C	0.001	18.199
Tajima D	-1.71*	0.111
Fu and Li's D*	-2.71*	0.95

Haplotypes are divided into two haplogroups (N284 and K284) based on the SNP (G/T) 1822 responsible for the K284N substitution. π = nucleotide diversity per site, θ = Watterson's estimator; *C* = recombination parameter; *= this value is significantly ($P < 0.05$) different from its neutral expectation based on the critical values obtained after coalescent simulations.
