# Supplementary material for: A candidate gene association study on muscat flavor in grapevine (Vitis vinifera L.)
Source: BMC Plant Biol. 2010 Nov 9;10:241. doi: 10.1186/1471-2229-10-241 (PMC3095323; doi:10.1186/1471-2229-10-241)
Supplement: Additional file 3 — List of the V. vinifera L. accessions analyzed in this study. 1 = neutral; 2 = aromatic; 3 = muscat flavor; § = unstable or slightly flavored; # = muscat-like aromatic mutants; * = neutral Muscats; Ŧ = FEM-IASMA accession; K284N = replacement of a Lysine with an Asparagine at site 284 caused by the SNP 1822. [file 1471-2229-10-241-S3.PDF]

Additional file 3.pdf

List of the *V. vinifera* L. accessions analyzed in this study.

Variety name	Variety number	Aroma phenotype	K284N substitution
A 406 SRLH	1211	3	N
Ag isioum	1563	1	K
Ak ouzioum tagapskii	2897	1	K
Albalonga = Würzburg B51-2-1	2700	2	K
Aleatico	194	3§	N
Alnwickseedling	755	2§	K
Annamaria	582	3§	N
Araklinos	1805	1	K
Armenia	2267	1	K
Aromatica =Pirovano 393	1051	3§	N
Aromriesling	601	2	K
Arpad = Eveque Prohaszka	1080	3§	N
Ascott Citronelle	721	3	N
Assario (2)	575	1*	K
Assyl kara	2505	1	K
Attila	751	3	N
Bakarka	3008	1	K
Barone dell'Aterno = Pirovano 184	995	3	N
Basilicumtraube	1600	2§	K
Bayadi du Liban	2998	1	K
Biczoegon	2534	3§	N
Boriska	2561	3	N
Bouquet Sylvaner	618	2§	K
Bouquettraube	281	2§	K
Brachetto	1242	3	N
Bruni 74	1129	2	N
Bruni 75	1130	2	N
Cabernet franc	324	1	K
Catanese nero	2398	1	K
César	225	1	K
Chardonnay 130	Commercial clone 130	1Ŧ	K
Chardonnay musqué Dijon 44-60	200	2#	K
Chasselas	585	1	K
Chasselas musqué	586	2#	K
Chirai obak	1186	1	K
Chouchillon	192	1	K
Daranyi Ignac	2439	3	N
David = Pirovano 109	967	3	N
DeliziadiVaprio = Pirovano 46a	908	3	N
Duchess of Buccleugh	692	3	N
Early Muscat	1970	3	N
Espadeiro tinto	1498	1	K
Exalta	2961	3	N

Feinriesling	606	2	N
Frappato di Vittoria	1318	1	K
Frühe Meraner	3183	1	K
Frühmuscat Oberlin	621	2	K
General de la Marmora faux	820	3§	K
Gewürztraminer	258	2#	K
Gloria Hungariae	2138	1*	K
Gold	2613	3	N
Ida	1418	3§	N
Impero =Pirovano 264	1017	2§	N
Irsay Oliver	1448	3	N
Isa = INRA 2012-55	2978	3§	N
Italia = Pirovano 65	926	3§	N
Jo Rizling	2563	2	K
July Muscat	2282	3	N
Kapistoni tétri	3242	1	K
Katta-kourgan	556	1	K
Kichmich tcherni	3264	1	K
Kisilovy	3349	1	K
Krakhouna	2638	1	K
Lameiro	3380	1	K
Lumassina	3312	1	K
Lutea = Pirovano 167	990	3	N
Malaga II	2570	3	N
Malvasia di Sardegna	2166	1	K
Malvasia di Candia aromatica	2382	3	N
Malvasia Moscatel Fontegrande	1176	1*	K
Mariensteiner = Würzburg B51-7-3	2699	2	K
Mathiasz Janosne	1077	3	N
Médouar	3381	1	K
Mehdik	2082	1	K
Melbento	1172	3	N
Mireille MI 108 C	2713	1	N
Misguli kara	2917	1	K
Montedoro = Pirovano 240	1012	3	N
Moriot Muskat = Geilweilerhof I-28-30	2728	3	N
Moscatel de Camarate	1173	3§	N
Moscatel Dr Soares Franco	1175	3	N
Moscatel rosado	2120	3	N
Moscato giallo	569	3	N
MoscatoTiammor = Pirovano 672	2337	3§	N
Mouchketny	2739	3§	K
Mourisco (Coll. EVV Amandio Galhano)	3379	1	K
Munkatsy Jozsef Muskotaly	2256	3§	N
Muscat à petit grain	555	3	N
Muscat Bifere	561	3	N
Muskat blume	615	2§	N
Muscat d'Alexandrie	308	3Ŧ	N
Muscat de Colmar	2975	3	N
Muscat d'Eisenstadt	687	1§	N
Muscat d'Hambourg (= lady Hasting)	584	3	N
Muscat d'Istamboul	398	3	N
Muscat d'Ouzbekistan	2647	3	N
Muscat de Rousse	2963	3	N
Muscat de Saumur	2610	2§	K
Muscat deTerracina	577	3	N

Muscat fleur d'oranger	570	3	N
Muscat fleur d'oranger faux	571	3	N
Muscat gris de la Calmette	540	3	N
Muscat Laserelle	685	1*	K
Muscat Lierval	679	1*	N
Muscat Madresfield Court	690	3§	N
Muscat Mikveh	1171	3§	N
Muscat Oberlin	620	2§	K
Muscat Ottonel	280	3	N
Muscat rouge de Madere	576	3	N
Muscat Saint Laurent	680	3	N
Muscat Vira	2646	3	N
Noir Glady	805	2§	N
Noir hâtif de Marseille	684	3§	N
Nuno gomes	2257	3§	N
Onusta	1980	1	K
Orangetraube	1569	2	K
Orbois	294	1	K
Org tokos	573	1§	N
Orlovi nogti	2461	1	K
Örrökké piros	2567	2§	K
Pecsi Szagos	1588	3	N
Perle de Csaba	1069	3	N
Pervenetz praskoveisky	2651	1	K
Pinces Black	689	3§	N
Pirovano 190	1225	3	N
Plant du Maroc E (Coll. Meknès)	2158	1	K
Pletchistik	2652	1	K
Portan	2796	1	K
Raisin banane noir	3384	1	K
Retagliado bianco	67	1	K
Riesling bleu	3073	1	K
Savagnin	N 867 B 25-4	1Ŧ	K
Siegerrebe = Scheu 7957	1196	2§	K
Tandanya faux	3279	1	K
Taschly	1182	3	K
Teresita = Dalmasso XVIII-24	1230	3§	N
Thallocsy Lajos muskotaly	1087	3§	N
Tinto Cao	1488	1	K
Tsitsa Kaprei	2471	1	K
Torrontes riojano	2425	3	N
Trollinger Muskat	2692	3§	N
Tsolikouri	2668	1	K
Uburebekur	3270	1	K
Variété d'oasis Bou Chemma 46	3281	1	K
Veltliner rot	284	1	K
Volta = Pirovano 105	963	3§	N
Voskeat	2511	1	K
Yapincack faux	3292	1	K

1 = neutral; 2 = aromatic; 3 = muscat flavor; § = unstable or slightly flavored; # = muscat-like aromatic mutants; * = neutral Muscats; Ŧ = FEM-IASMA accession; K284N = replacement of a Lysine with an Asparagine at site 284 caused by the SNP 1822.
