# Supplementary material for: A candidate gene association study on muscat flavor in grapevine (Vitis vinifera L.)
Source: BMC Plant Biol. 2010 Nov 9;10:241. doi: 10.1186/1471-2229-10-241 (PMC3095323; doi:10.1186/1471-2229-10-241)
Supplement: Additional file 4 — List of primers used in colony PCR and cloned cDNA VvDXS allele sequencing. [file 1471-2229-10-241-S4.PDF]

Additional file 4.pdf

List of primers used in colony PCR and cloned cDNA *VvDXS* allele sequencing.

Primer name	Sequence (5’-3’)
M13fw	GTAAAACGACGGCCAGT
ex_dxs_2rw	GAAGAGATTCAAGCCCGTT
ex_dxs_2fw	CACAGTTCTACTACCATCTCAGC
ex_dxs_3rw	GCGACTGTTATTCGTAAGCCA
ex_dxs_3fw	AGAAGCAGAGGTGGACAAGG
ex_dxs_4fw	GGCTATGGAACAGCAGTACAGAG
M13rw	CAGGAAACAGCTATGAC
