# Supplementary material for: A candidate gene association study on muscat flavor in grapevine (Vitis vinifera L.)
Source: BMC Plant Biol. 2010 Nov 9;10:241. doi: 10.1186/1471-2229-10-241 (PMC3095323; doi:10.1186/1471-2229-10-241)
Supplement: Additional file 5 — List of primers used for VvDXS genomic sequencing. [file 1471-2229-10-241-S5.PDF]

Additional file 5.pdf

List of primers used for *VvDXS* genomic sequencing.

Amplicon	Primer name	Sequence (5'-3')
1	DXS6fw	ATGGCTCTCTGTACGCTCTCA
	DXS6rw	GTGTGGGTAAGACTTCAGAAACA
2	DXS7fw	GGTTACAATCTCACCTTCTCTG
	DXS7rw	CATCACCTATGACAGCAATGAC
3	DXS8fw	CAACAACGTCATTGCTGTCATAG
	DXS8rw	GCTAGACAGAACAGGTAAGATTTC
4	DXS9fw	CCAAACAGATTGGCGGACCG
	DXS9rw	CATAGCATTTGATTAAGAAGATATGGT
5	DXS10fw	CAGAAGCAGAGGTGGACAA
	DXS10rw	CGGACACTAAGGTCATAGGCT
6	DXS11fw	CAGCCACTTGTCTCATTGTG
	DXS11rw	CTCCAACCAGCCCAGC
7	DXS12fw	ATAGCGTTAGTTGGAAAACCG
	DXS12rw	ATTGACCCTTCTTCTACTGTAATCA
8	DXSpolyAfw	GAACAACATGGCTTACGAATAAC
	DXSpolyArw	TCCTATCATGGCATCCTTTC
